# Investigation of Temperature-Dependent Magnetic Properties and Coefficient of Thermal Expansion in Invar Alloys

**DOI:** 10.3390/ma15041504

**Published:** 2022-02-17

**Authors:** Lin Huang, Yongjian Zhou, Tingwen Guo, Dong Han, Yu Gu, Cheng Song, Feng Pan

**Affiliations:** 1 Key Laboratory of Advanced Materials (MOE), School of Materials Science and Engineering, Tsinghua University, Beijing 100084, China; zhouyj18@mails.tsinghua.edu.cn (Y.Z.); gtw18@mails.tsinghua.edu.cn (T.G.); songcheng@mail.tsinghua.edu.cn (C.S.); panf@mail.tsinghua.edu.cn (F.P.); 2 Shanxi Taigang Stainless Steel Co., Ltd., Taiyuan 030003, China; handong@tisco.com.cn (D.H.); guyu01@tisco.com.cn (Y.G.)

**Keywords:** invar alloy, coefficient of thermal expansion, domain structure, magnetostriction

## Abstract

Invar Fe–Ni alloy is a prominent Ni steel alloy with a low coefficient of thermal expansion around room temperature. We investigate the correlation between magnetic properties and thermal expansion in cold-drawn Fe–36Ni wires with different heat treatment conditions, where the annealing parameters with furnace cooling (cooling from the annealing temperature of 300, 400, 500, 600, 700, 800, 900, and 1000 °C) are used. The variation trend of magnetic properties is consistent with that of thermal expansion for all samples, where the maximum appears at 600 °C -treated sample and 400 °C shows the minimum. The domain size and the area of domain walls determine the total energy of the domain wall, and the total energy directly determines the size of magnetostriction, which is closely related to the coefficient of thermal expansion. Also, the differential thermal analysis (DTA) shows endothermic and exothermic reactions represent crystalline transitions, which could possibly cause the abrupt change of magnetic properties and thermal expansion coefficient of materials. The results indicate that there is a certain relation between thermal expansion and magnetic properties. Besides the fundamental significance, our work provides an Invar alloy with a low coefficient of thermal expansion for practical use.

## 1. Introduction

In 1897, Guillaume found a very low coefficient of expansion (0.877 × 10^−6^/°C) in 36% Ni–Fe alloys, which did not completely conform to the normal thermal expansion law. It was an abnormal thermal expansion phenomenon and was called Invar alloy [1,2]. The factors affecting the thermal expansion coefficient of Invar alloys mainly include alloy composition, grain size, defect content, and precipitation. The Invar concentration 35.7% Ni–Fe (mass fraction) alloy shows the lowest thermal expansion coefficient, and the thermal expansion coefficient increases significantly regardless of the change of Ni or Fe content on both sides of the composition. Therefore, accurate control of Ni content is the key to obtain the Invar alloy [3]. It also has been reported that the internal defects of Ni–Fe alloys increase and the density decreases due to cold machining, which destroys the degree of short-range atomic ordering and affects the spontaneous magnetization and magnetostriction system of the alloys. Finally, the thermal expansion coefficient of the alloys is reduced, and even becomes negative [4]. Invar alloys also exhibit particular magnetic properties such as sudden deviation of spontaneous magnetization, the large high susceptibility, strong pressure-dependence of the Curie temperature (*T_c_*), and an easy demagnetization [5,6,7]. The phenomena above are generally considered as Invar effects. Invar alloys are widely used in aeronautics and astronautics, motor valves, seismic creep, precision instruments, etc. [8,9,10]. Thus, Invar alloys have attracted extensive attention with increasing interest [11,12,13]. 

In general, a common material reflects a nature of thermal expansion and cold contraction, showing a normally large coefficient of thermal expansion [14]. Differently, Invar alloys always show an abnormally small coefficient of thermal expansion, which is commonly explained by the specific changes in their micro-structure [15], the formation of a short-range or long-range order, and of a modulated structure [16,17,18]. Alternatively, the low coefficient of thermal expansion of Invar alloys may also be caused by the fact that the normal lattice variation is compensated by the robust spontaneous volume magnetostriction in a wide temperature range [9,19,20]. However, how these magnetic properties systematically affect α is still unknown. The experiments below investigate the correlation between magnetic properties and the coefficient of thermal expansion in cold-drawn Fe–36Ni wires with different heat treatment conditions. From our results, we found that the trend of thermal expansion is consistent with that of saturation magnetization and energy density of magnetic domain determines the size of magnetostriction with different heat treatment conditions. Moreover, the differential thermal analysis (DTA) shows endothermic and exothermic reactions represent crystalline transitions, which indicates the abrupt change of magnetic properties and thermal expansion coefficient of materials. 

## 2. Experimental Section

The materials used in this work are cold-drawn Invar Fe–36Ni alloy wires prepared by Shanxi Taigang Stainless Steel Co., Ltd., Shanxi, China, with a diameter range of 2–4 mm. The chemical composition of raw sample is shown in Table 1. Based on the composition, it is a typical Fe–36Ni alloy (hereafter referred to as raw sample). Invar alloy wires were annealed at different temperatures (*T*) of 300 (sample T300), 400 (T400), 500 (T500), 600 (T600), 700 (T700), 800 (T800), 900 (T900), and 1000 °C (T1000) for 2 h and then underwent furnace cooling. Magnetic properties were characterized by vibrating sample magnetometer (VSM) (LakeShore 8604 Westerville, OH, USA), and magnetic force microscope (MFM). The differential thermal analysis (DTA) was measured by thermogravimetric-differential thermal analysis (No.STA499F3 NETZSCH-Gerätebau GmbH, Selb, Germany). The coefficients of thermal expansion were measured by a thermal analysis system (No.DIL805ADT, New Castle, DE, USA), and the coefficient of thermal expansion value α was the average value of measured temperature range form 20–100 °C (heating start temperature was 15 °C and end temperature was 120 °C), the temperature ramp was 5 °C/min. Moreover, we used cylindrical samples with a diameter of 4 mm and length of 10 mm; each sample was repeated three times to extract the coefficient of thermal expansion. 

## 3. Results and Discussion 

### 3.1. VSM Studies

**Figure 1:**
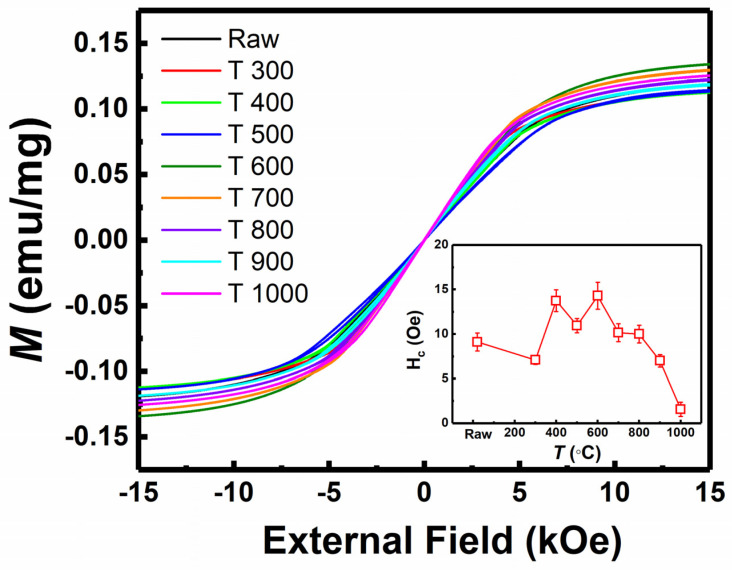
*M-H* loops of Invar alloy wires with various heat treatment conditions (raw, T300, T400, T500, T600, T700, T800, T900, T1000), respectively. And *H_c_* as a function of heat treatment temperature (*T*) extracted from *M–H* loops is shown in the inset.

In the following experiments, we mainly focus on the influence of magnetic properties of Invar alloy on thermal expansion coefficient. After heat treatment at different temperatures, the relationship between magnetic variation on magnetostriction and thermal expansion coefficient is obtained. The magnetic properties of Invar alloy wires with different heat treatment conditions are investigated. The perpendicular magnetic hysteresis loops (*M-H* loops) in Figure 1 are measured at room temperature. We repeat the measurement with three samples to extract the saturation magnetization *M_s_*. All of the *M-H* loops demonstrate strong soft magnetic properties with small coercive field (*H_c_*) (smaller than 15 Oe). *H_c_* is extracted from the *M-H* loops and samples of annealing temperature at 400 °C and 600 °C show almost the same *H_c_* and higher than others. However, the saturation magnetization (*M_s_*) with different annealing temperatures shows a significantly different trend, which affects the variation trend of thermal expansion coefficient, as we will explain later. 

### 3.2. Comparison of Thermal Expansion Coefficient α and Saturation Magnetization M_s_

**Figure 2:**
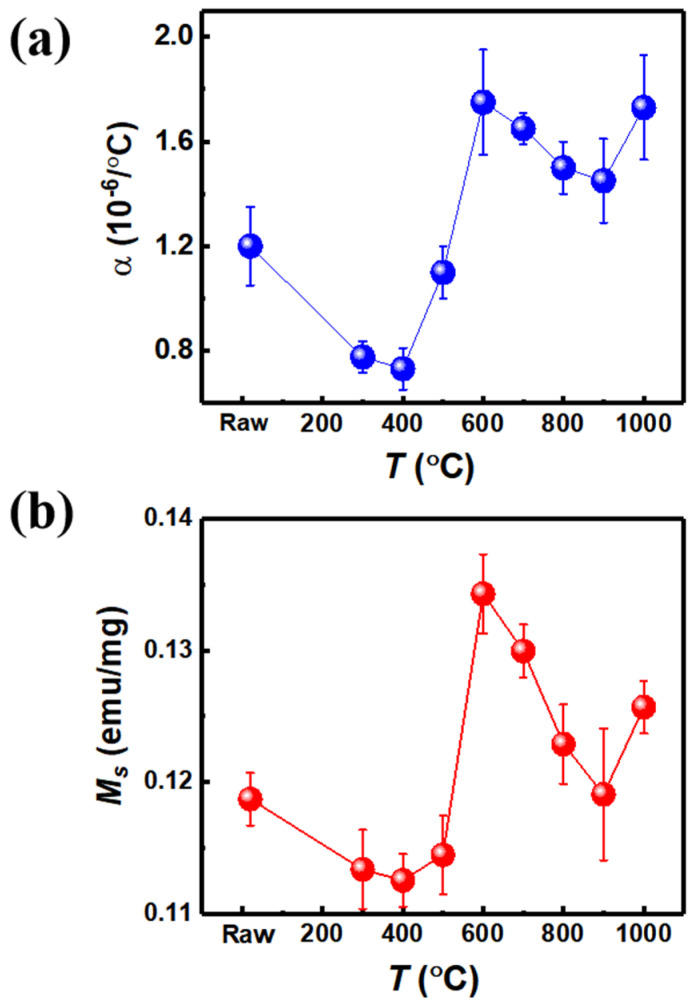
(**a**) The coefficient of thermal expansion (*α*) as the function of the heat treatment temperature (*T*). (**b**) Saturation magnetization *M_s_* as a function of heat treatment temperature (*T*) extracted from *M–H* loops.

The coefficient of thermal expansion *α* as a function of heat treatment temperature is shown in Figure 2a. Wherein, the coefficient of thermal expansion value α is the average value of measured temperature range form 20–100 °C (heating start temperature is 15 °C and end temperature is 120 °C), the temperature ramp is 5 °C/min. Moreover, we used the cylindrical samples with a diameter of 4 mm and length of 10 mm; each sample was repeated three times to extract the coefficient of thermal expansion. In particular, with the increasing of annealing temperature, the change of thermal expansion coefficient does not show a linear trend, it is similar with the trend of saturation magnetization *M_s_* (Figure 2b). At the heat treatment, condition T400 shows the lowest *M_s_* (0.1125 emu/mg) and coefficient of thermal expansion value α (0.73 × 10^−6^/°C); T600 shows the highest *M_s_* (0.1342 emu/mg) and the coefficient of thermal expansion value α (1.75 × 10^−6^/°C). The details of Invar alloy wires about different heat treatment conditions, XRD phases, *M_s_*, and α are summarized in Table 2. It is worth noting that the α value of T600 is much higher than T400, which indicates the magnetic properties play a role for the coefficient of thermal expansion for Invar alloy wires. It is indicated that the thermal expansion value α is proportional to the *M_s_*_,_ and to the α∝∆L/L value, which is as well as spontaneous volume magnetostriction *λ.* The fact that we can estimate that the spontaneous volume magnetosriction *λ* is proportional to *M_s_* is consistent with the research, as Ref. [21] showed. The spontaneous volume magnetostriction *λ* is proportional to the square of the magnetization *M^2^* as: $\lambda ={\kappa C}^{\mathrm{band}}M^{2}$, where C^band^ is the magnetovolume couping constant [21]. 

### 3.3. MFM Studies

**Figure 3:**
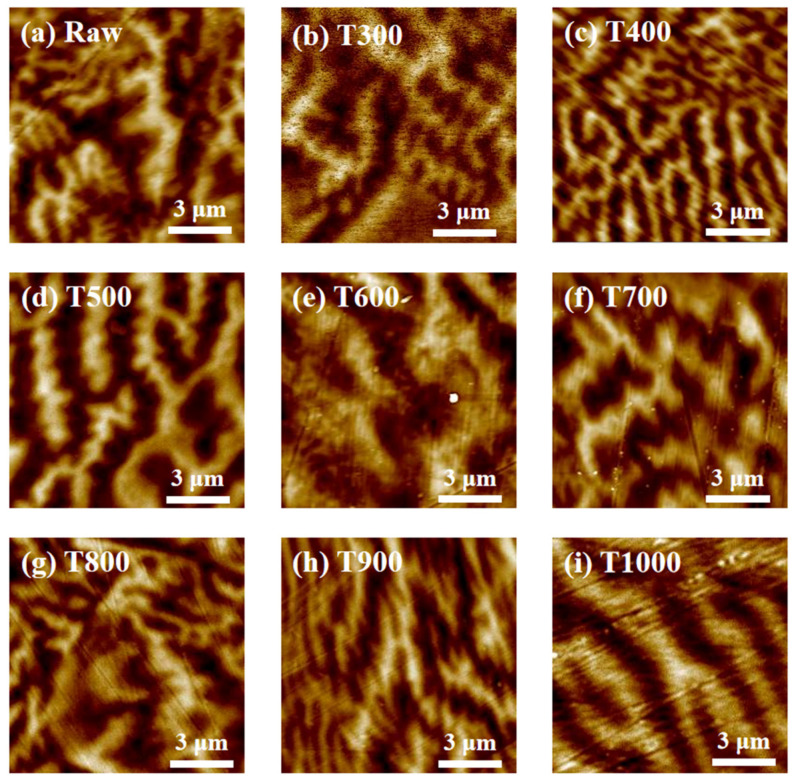
Magnetic domain images of raw (**a**), T300 (**b**), T400 (**c**), T500 (**d**), T600 (**e**), T700 (**f**), T800 (**g**), T900 (**h**), and T1000 (**i**), at room temperature.

Furthermore, the magnetic domain shows the direct evidence which verifies the effect of magnetic properties on coefficient of thermal expansion. MFM images measured at room temperature are shown in Figure 3, raw, T300, T400, T500, T600, T700, T800, T900, T1000 correspond to Figure 3a–i, respectively. However, as the heat treatment temperature varies, the total energy *f* = *ε* S (*T*) [22] of the domain walls changes, where *ε* is the domain wall energy density and S (*T*) is the domain wall area of samples with annealing temperature *T*. Note that MFM could only detect out of plane magnetization, thus all black and white magnetic domains are 180 degrees, which are antiparallel to each other. In the equilibrium structure, the relationship between the domain wall energy density *ε* and domain wall bending degree R is: ε∝R. Among all samples, the domain wall area of the T400 sample is the largest and the degree of bending is highest, while that of T600 is the smallest. It indicates the highest total energy of domain walls *f* and domain wall energy density *ε* with T400, and lowest with T600. The relationship between the initial magnetic susceptibility *χ_i_* of Invar alloys and the energy density of the domain walls is $\chi_{i}\;\propto \frac{M_{s}^{2}}{\varepsilon }$ [22], which results in a minimum *χ_i_* value of T400 and maximum of T600. According to the above analysis, the change of material magnetostriction *λ* can be expressed as: $\lambda \;\propto \;\frac{1}{\chi_{i}}\;\propto \varepsilon$. Additionally, it can be estimated that the area of domain S is inversely proportional to the *λ*, and the results shown in Figure 2 are also supporting this opinion. Therefore, according to previous discussions, the material magnetostriction *λ* of samples can be defined as *λ*_T400_ > *λ*_(Raw, T300, T500, T700, T800, T900, T1000)_ > *λ*_T600._ T400 obtains the highest magnetostriction and T600 the lowest. Magnetostriction is a change in volume, size or shape caused by a change in the magnetization state of a ferromagnet. Therefore, it is of practical significance to utilize and control this change in the study of Invar alloys. When bending of the magnetic domain is large, the magnetostriction is relatively large, then the magnetic expansion can effectively eliminate the normal lattice expansion, resulting in a very low coefficient of thermal expansion. Conversely, a high coefficient of thermal expansion can be obtained if magnetostriction is weak. Therefore, the highest coefficient of thermal expansion *α* is shown in T600 and lowest *α* in T400. The above well explains the relationship between the change of thermal expansion coefficient and magnetic properties. 

### 3.4. DTA Studies

**Figure 4:**
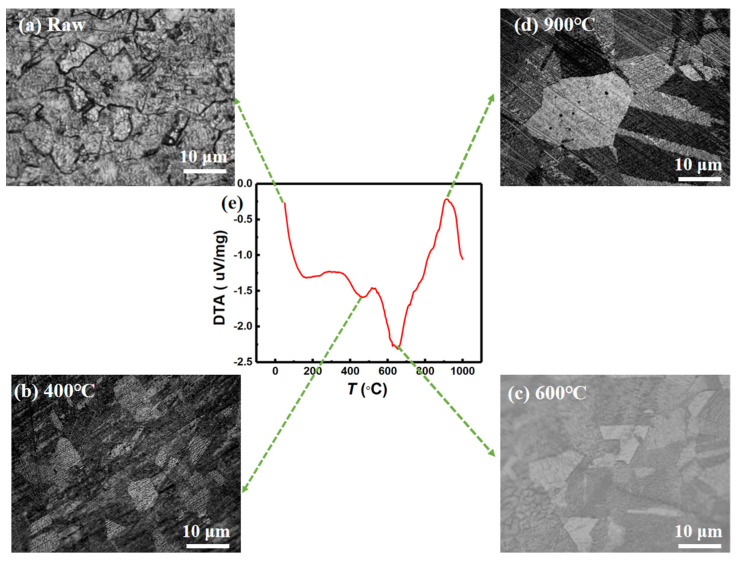
Micrograin structure of samples treated with different heat treatment processes: (**a**) raw, (**b**) 400 °C, (**c**) 600 °C, and (**d**) 900 °C. (**e**) Differential thermal analysis (DTA) as the function of temperature (range from 50 °C to 1000 °C).

Moreover, the differential thermal analysis (DTA) measured by thermogravimetric-differential thermal analysis (TG-DTA) in an inert atmosphere of argon, at the temperature range from 50 °C to 1000 °C, is plotted in Figure 4e. It shows the change of crystallization in invar alloy samples with increasing temperature. There is an obvious endothermic peak near 600 °C, and the phenomenon of endothermic peak is accompanied by the crystal transformation of alloy material. There is also a relatively small endothermic peak near 400 °C, and a clear exothermic peak at 900 °C, which is an exothermic reaction. Both endothermic and exothermic reactions represent the crystalline state of samples. After holding at 400 °C for 2 h annealing (Figure 4b), the recovery process was basically completed, that is, the storage energy caused by deformation was released. With the increase of annealing temperature 600 °C (Figure 4c), the grains grew bigger, indicating their recrystallization. When the annealing temperature is increased to 900 °C (Figure 4d), the grain coarsening is obvious. It is known that when the shape variable is constant, the higher the temperature, the larger the grain grows. We noted that the endothermic and exothermic peaks correspond to the changes of trends of saturation magnetization and thermal expansion.

From the above experimental results, it is known more clearly that the change of magnetic properties of the Invar alloy has a definite influence on the change of magnetostriction and thermal expansion. The coefficient of thermal expansion is proportional to magnetostriction *λ* and the square of the magnetization *M^2^*, but inversely proportional to the area of the magnetic domain S. This obvious change in magnetic properties allows us to provide more methods and a more comprehensive perspective on the size of the thermal expansion coefficient. 

## 4. Conclusions

We have investigated the effect of magnetic properties on the coefficient of thermal expansion with different heat treatment conditions for cold-drawn Fe–36Ni Invar alloy wires. By comparing the magnetic properties of the samples, the variation trend of all samples is consistent with that of the thermal expansion coefficient, T600 shows the maximum and T400 shows the minimum. The domain size and the area of domain walls determines the total energy of the domain wall, and the size of the energy directly determines the size of the magnetostriction, which is closely related to the coefficient of thermal expansion. Magnetostriction can effectively eliminate the normal lattice expansion, thus resulting in a low coefficient of thermal expansion. Moreover, the differential thermal analysis (DTA) shows endothermic and exothermic reactions represent crystalline transitions, which indicates the abrupt change of magnetic properties and thermal expansion coefficient of materials. The results indicate that there is a certain relation between thermal expansion and magnetic properties. Besides the fundamental significance, our work provides an Invar alloy with a low coefficient of thermal expansion for practical use. This obvious change in magnetic properties allows us to provide more methods and a more comprehensive perspective for estimating the thermal expansion coefficient. 

By observing the relationship between the coefficient of thermal expansion and magnetic properties, we can know that magnetic property is one of the factors affecting the change of thermal expansion coefficient. It is believed that magnetostriction has a certain effect on the thermal expansion coefficient, which cancels out the normal lattice contraction, resulting in an almost zero expansion rate at room temperature. This can be useful for a preliminary determination of the magnitude of the thermal expansion coefficients with their high magnetostriction and domain structures, in Invar alloy application areas.

## Figures and Tables

**Table 1:** Composition of the cold-drawn Invar Fe–Ni alloy wire with wt% ≥ 0.01%.

C	Si	Mn	Al	Cu	Ni	Fe
0.02	0.15	0.29	0.016	0.01	35.71	63.79

**Table 2:** Different heat treatment conditions, XRD phase, saturation magnetization (*M_s_*), and coefficient of thermal expansion (*α*).

Heat Treatment Conditions	XRD Phase	*M_s_* (emu/mg)	α (10^−6^/°C)
Raw	fcc	0.1187	1.2
T300	fcc	0.1133	0.77
T400	fcc	0.1125	0.73
T500	fcc	0.1144	1.1
T600	fcc	0.1342	1.75
T700	fcc	0.1299	1.65
T800	fcc	0.1228	1.5
T900	fcc	0.1190	1.45
T1000	fcc	0.1256	1.73

## Data Availability

The data presented in this study are available on request from the corresponding author.
